# A randomized, controlled trial of ZYN002 cannabidiol transdermal gel in children and adolescents with fragile X syndrome (CONNECT-FX)

**DOI:** 10.1186/s11689-022-09466-6

**Published:** 2022-11-25

**Authors:** Elizabeth Berry-Kravis, Randi Hagerman, Dejan Budimirovic, Craig Erickson, Helen Heussler, Nicole Tartaglia, Jonathan Cohen, Flora Tassone, Thomas Dobbins, Elizabeth Merikle, Terri Sebree, Nancy Tich, Joseph M. Palumbo, Stephen O’Quinn

**Affiliations:** 1 Departments of Pediatrics and Neurological Sciences, Rush University Medical Center, Chicago, IL USA; 2 Medical Investigation of Neurodevelopmental Disorders (MIND) Institute, University of California-Davis Medical Center, Sacramento, CA USA; 3 Department of Pediatrics, University of California Davis School of Medicine, Sacramento, CA USA; 4 Departments of Psychiatry and Child Psychiatry, Fragile X Clinic, Kennedy Krieger Institute/the Johns Hopkins Medical Institutions, Baltimore, MD USA; 5 Department of Psychiatry & Behavioral Sciences-Child Psychiatry, Johns Hopkins School of Medicine, Baltimore, MD USA; 6 Department of Psychiatry and Behavioral Neuroscience, Cincinnati Children’s Hospital Medical Center and the University of Cincinnati College of Medicine, Cincinnati, OH USA; 7 Centre for Clinical Trials in Rare Neurodevelopmental Disorders, Children’s Health Queensland, Brisbane, Australia; 8 Centre for Child Health Research, University of Queensland, Brisbane, Australia; 9 Department of Pediatrics, Developmental Pediatrics, University of Colorado School of Medicine, Children’s Hospital Colorado, Aurora, CO USA; 10 Fragile X Alliance Inc, North Caulfield, VIC, Australia; 11 Centre for Developmental Disability Health Victoria, Monash University, Clayton, VIC Australia; 12 Department of Biochemistry and Molecular Medicine, School of Medicine, University of California-Davis, Sacramento, CA USA; 13 The Griesser Group, Conshohocken, PA USA; 14 Labcorp Drug Development, Durham, NC USA; 15 Zynerba Pharmaceuticals Inc., Devon, PA USA

**Keywords:** Fragile X syndrome, Clinical trial, Endocannabinoid system, Cannabidiol

## Abstract

**Background:**

Fragile X syndrome (FXS) is associated with dysregulated endocannabinoid signaling and may therefore respond to cannabidiol therapy.

**Design:**

CONNECT-FX was a double-blind, randomized phase 3 trial assessing efficacy and safety of ZYN002, transdermal cannabidiol gel, for the treatment of behavioral symptoms in children and adolescents with FXS.

**Methods:**

Patients were randomized to 12 weeks of ZYN002 (250 mg or 500 mg daily [weight-based]) or placebo, as add-on to standard of care. The primary endpoint assessed change in social avoidance (SA) measured by the Aberrant Behavior Checklist–Community Edition FXS (ABC-C_FXS_) SA subscale in a full cohort of patients with a FXS full mutation, regardless of the *FMR1* methylation status. Ad hoc analyses assessed efficacy in patients with ≥ 90% and 100% methylation of the promoter region of the *FMR1* gene, in whom *FMR1* gene silencing is most likely.

**Results:**

A total of 212 patients, mean age 9.7 years, 75% males, were enrolled. A total of 169 (79.7%) patients presented with ≥ 90% methylation of the *FMR1* promoter and full mutation of *FMR1*. Although statistical significance for the primary endpoint was not achieved in the full cohort, significant improvement was demonstrated in patients with ≥ 90% methylation of *FMR1* (nominal *P* = 0.020). This group also achieved statistically significant improvements in Caregiver Global Impression‐Change in SA and isolation, irritable and disruptive behaviors, and social interactions (nominal *P*-values: *P* = 0.038, *P* = 0.028, and *P* = 0.002). Similar results were seen in patients with 100% methylation of *FMR1*. ZYN002 was safe and well tolerated. All treatment-emergent adverse events (TEAEs) were mild or moderate. The most common treatment-related TEAE was application site pain (ZYN002: 6.4%; placebo: 1.0%).

**Conclusions:**

In CONNECT-FX, ZYN002 was well tolerated in patients with FXS and demonstrated evidence of efficacy with a favorable benefit risk relationship in patients with ≥ 90% methylation of the *FMR1* gene, in whom gene silencing is most likely, and the impact of FXS is typically most severe.

**Trial registration:**

The CONNECT-FX trial is registered on Clinicaltrials.gov (NCT03614663).

**Supplementary Information:**

The online version contains supplementary material available at 10.1186/s11689-022-09466-6.

## Introduction

### Fragile X syndrome

Fragile X syndrome (FXS) is a rare X-linked genetic disorder that has a prevalence of approximately 1 in 4000 males and 1 in 6000 females [1]. FXS is the most common inherited cause of intellectual disability and monogenetic cause of autism spectrum disorder (ASD) and is also associated with anxiety and a variety of problem behaviors such as aggression, irritability, temper tantrums, hyperactivity, attention deficits, shyness, and preference for solitary activities [2, 3]. Despite decades of preclinical research and interventional clinical trials, there are no regulatory approved treatments for FXS.

### FXS pathophysiology

FXS is typically caused by a trinucleotide repeat expansion containing more than 200 cytosine, guanine, and guanine (CGG) repeats in the 5′ untranslated region of the *FMR1* gene on the X chromosome (full mutation, [FM]). This generally leads to methylation of the promoter region of *FMR1*, producing subsequent gene silencing and absent or reduced levels of the protein product, FMRP [4–9]. Thus, FXS is caused by the deficiency or absence of FMRP [10].

FMRP is an RNA-binding protein that is important for normal synaptic function, synaptic plasticity, and the development of neuronal connections over time during brain maturation [11]. In general, the FXS cognitive and emotional phenotype depends on the amount of FMRP that is produced, which is determined in part by the degree of methylation of *FMR1* [9, 12]. Males with a fully methylated FM generally do not produce FMRP, while FMRP can range from near normal to significantly reduced in females with a fully methylated FM of *FMR1*, depending on the pattern of X-inactivation in the affected female [13, 14]. In general, patients with a higher degree of methylation have a more severe phenotype, including lower IQ and more severe symptoms of ASD, although there is wide variability for any given level of methylation [14]. Some individuals with FXS are mosaics and can present with a high degree of mosaicism due to the presence of a percentage of cells with the *FMR1* premutation (i.e., 55 to 200 CGG repeats) in addition to cells with a full mutation. They can also present with incomplete methylation and can produce elevated *FMR1* mRNA, which can cause toxicity to the cells of the central nervous system (CNS). Those with > 90% methylation produce lower levels of *FMR1* mRNA and little or no FMRP. Therefore, they present the classical and most severe phenotype of FXS that is recapitulated by the knockout mouse model of FXS.

### Endocannabinoid system is dysregulated in FXS

The endocannabinoid (EC) system includes 2 types of G-protein–coupled receptors, termed CB_1_ and CB_2_ [15, 16]. Cannabinoid receptors are found in a variety of diverse organisms [17], indicating that the EC system is highly evolutionarily conserved and may play central roles in human physiology and pathophysiology [18]. CB_1_ receptors are among the most abundant G-protein–coupled receptors in the brain and are present at lower concentrations in a variety of peripheral tissues and cells [19]. CB_2_ receptors are expressed primarily in the immune and hematopoietic systems, as well as in the brain, pancreas, and bone.

The primary endogenous ligands for CB_1_ and CB_2_ receptors are called ECs and include anandamide (AEA) and 2-arachidonoylglycerol (2-AG). The ECs modulate synaptic transmission throughout the CNS, yielding widespread influence on cognition and behavior [20, 21]. The ECs are synthesized and released “on demand” from post-synaptic membrane-bound phospholipids in response to neuronal signaling and act as retrograde signaling molecules across the synaptic cleft to stimulate CB_1_ receptors on the presynaptic terminal [15, 22] and attenuate further activity through an inhibitory feedback loop. Enzymes that function in synthesizing 2-AG include phospholipase C and diacylglycerol lipase (DAGL) [15, 23]. At developed synapses, 2-AG released from postsynaptic terminals binds to presynaptic CB_1_ receptors to inhibit the secretion of both excitatory and inhibitory neurotransmitters; this DAGL-dependent synaptic plasticity operates throughout the nervous system [24].

The functional consequences of reduced FMRP in FXS likely reflect changes in both developmental and dynamic regulation of multiple intracellular processes. Among these, loss of FMRP function is thought to alter DAGL function and retrograde EC signaling in neuronal synapses, thereby affecting excitatory and inhibitory neurotransmitter release [24]. Downstream dysregulation of EC signaling in the CNS is one proposed mechanism that may contribute to the clinical abnormalities seen in FXS [24, 25].

Cannabidiol, the main non-euphoric component of the Cannabis plant, has a variety of effects on the EC system that may improve the behavioral symptoms of FXS. These include, (A) attenuating the loss of endogenous, on-demand EC signaling by increasing 2-AG availability and serving as retrograde signaling molecules in the regulation of synaptic transmission through negative allosteric modulation of the CB_1_ receptor [24, 26, 27]; (B) preventing internalization of CB_1_ receptors and restoring membrane expression of receptors [28–32]; and (C) increasing the availability of AEA by reducing its access to the catabolic pathway [26, 31, 33–36].

Cannabidiol may also have other effects related to FXS. Cannabidiol binds to the 5HT_1A_ receptor with moderate affinity and possesses agonist efficacy in 5HT_1A_ signal transduction studies [37]. Cannabidiol has also been shown to act as a positive allosteric modulator at GABA_A_ receptors [38]. Cannabidiol’s ability to enhance EC levels and facilitate GABAergic transmission may serve to improve the balance in inhibitory and excitatory transmission and help restore neuronal function and synaptic plasticity in patients with FXS. Cannabidiol is also a dopamine partial agonist [39].

### Rationale for the CONNECT-FX trial

ZYN002 is a permeation-enhanced transdermal gel, consisting of a hydro-alcoholic gel containing cannabidiol, which is manufactured at a 4.2% (w/w) cannabidiol concentration. An exploratory phase 2 open-label clinical trial (ZYN2-CL-009) found that 12-week treatment with ZYN002 resulted in clinically meaningful reductions in anxiety and behavioral symptoms in 20 children and adolescents with FXS and full *FMR1* mutation [40]. In light of these findings, the placebo-controlled, phase 3, CONNECT-FX trial was conducted to assess the efficacy and safety of ZYN002 for the treatment of behavioral symptoms in children and adolescents with FXS.

## Methods

### Study design

**Fig. 1:**
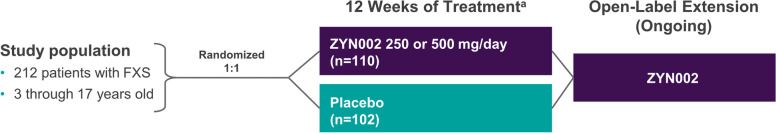
Study design. ^a ^indicates the following: following a 2-week single-blind placebo run-in period

CONNECT-FX (Study ZYN2-CL-016) was a randomized, double-blind, placebo-controlled efficacy and safety study in pediatric and adolescent patients with FXS aged 3 to < 18 years. The study design for the CONNECT-FX trial is shown in Fig. 1. Patients with FXS were treated for 12 weeks with a 2-week, single-blind placebo run-in preceding the 12-week double-blind treatment period. Following the placebo run-in period, patients who met the following criteria were randomized 1:1 to receive ZYN002 or placebo: ABC-C_FXS_ Social Avoidance (SA) score ≥ 4 prior to randomization, with no more than a 30% improvement during the placebo run-in, or ABC-C_FXS_ SA score of 2 or 3 with an ABC-C_FXS_ Irritability (Irr) score ≥ 18 prior to randomization, with no more than a 30% improvement in ABC-C_FXS_ SA score during the placebo run-in. Randomization was stratified by gender, weight category, and region.

Dose selection for this trial was based upon findings from the initial open-label trial in patients with FXS in which the majority of individuals were titrated up to 250 mg/day [40]. The open-label data demonstrated a potential efficacy signal with good tolerability. These findings led to the selection of doses of 250 mg/day or 500 mg/day for individuals ≤ 35 kg or > 35 kg respectively in CONNECT-FX, supported by population pharmacokinetic modeling to predicted dosing to provide similar steady-state concentrations found in adults at a dose of 500 mg/day which had been demonstrated to be safe and well tolerated. ZYN002 was provided in sealed individual dose packets containing 125 mg cannabidiol per individual packet and placebo packets matched the study drug dosing packets in appearance and gel contents (without cannabidiol). In a blinded fashion, ZYN002-treated patients who weighed ≤ 35 kg received 125 mg cannabidiol Q12H (1 individual dose packet every 12 h) (± 2 h), for a total daily dose of 250 mg cannabidiol. Patients who weighed > 35 kg received 250 mg cannabidiol (2 dose packets) Q12H (± 2 h), for a total daily dose of 500 mg cannabidiol. All patients remained on their assigned dose during the 12 weeks of the treatment phase of the study. Study visits occurred at week 4, week 8, and week 12 post randomization. Patients who successfully completed the 12 weeks of the double-blind study and were at least 90% compliant with the trial drug and visits had the option to enroll in an open-label extension study.

### Study population

The trial was conducted at 21 investigative centers in the USA (17 sites), Australia (3 sites), and New Zealand (1 site). Children and adolescents aged 3 to < 18 years with a body mass index of 12–30 kg/m^2^ and a diagnosis of FXS through molecular documentation of the full *FMR1* mutation were enrolled. At screening, all patients were required to have an ABC-C_FXS_ SA subscale score ≥ 4 or an ABC C_FXS_ SA subscale score of 2 or 3 with an ABC-C_FXS_ Irr subscale score ≥ 18. All patients were also required to have a Clinical Global Impression-Severity (CGI-S) score ≥ 3. Patients who were receiving psychotropic medication(s) were eligible provided they were taking ≤ 2 medications for ≥ 4 weeks prior to enrollment. Patients with a history of seizure disorders who were receiving ≤ 2 antiseizure medications or who were seizure-free for ≥ 1 year prior to the study were eligible. Key exclusion criteria were as follows: use of cannabis or any tetrahydrocannabinol (THC) or cannabidiol-containing product within 3 months of study entry or during the study; alanine aminotransferase, aspartate aminotransferase, or total bilirubin levels ≥ 2 × upper limit of normal (ULN) or alkaline phosphatase levels ≥ 3 × ULN; positive drug screen (ethanol, cocaine, THC, barbiturates, amphetamines [unless prescribed], benzodiazepines [except midazolam], and opiates); use of the following antiepileptic drugs: clobazam, phenobarbital, ethosuximide, felbamate, or vigabatrin; and use of a strong inhibitor/inducer of cytochrome P450 3A4.

Patients were randomly assigned to treatment according to a computer-generated randomization scheme. Randomization was coordinated centrally through an interactive response system (IRT). Patients who met all eligibility requirements and randomization criteria were randomly allocated to active or placebo treatment groups using a 1:1 allocation ratio. Randomization was stratified by sex (male vs female), weight (≤ 35 kg vs > 35 kg), and region (North America vs non-North America).

### CGG repeat sizing and methylation status

Genomic DNA was isolated from peripheral blood leukocytes using Gentra Puregene Blood Kit (Qiagen, Valencia, CA). Molecular DNA testing for FXS, to establish CGG repeat length, percentage of CpG methylated *FMR1* alleles, and, in females, activation ratio was carried out by PCR and Southern blot analysis as previously described [41–43].

### Outcome measures

The primary efficacy end point was the change from baseline (day 1 of randomized treatment) to week 12 in the ABC-C_FXS_ SA subscale score. Key secondary end points included change from baseline to week 12 in ABC-C_FXS_ Irr subscale score, change from baseline to week 12 in ABC-C_FXS_ Social Unresponsiveness/Lethargy (SU/L) subscale score, and Clinical Global Impression, Improvement (CGI-I) at week 12. Exploratory end points to support meaningful change analyses included domain specific (SA, Irr, and SU/L) and overall behavior Caregiver Global Impression of Severity and Change items (CaGI-S and CaGI-C).

The ABC-C is an established observer-reported outcome measure of inappropriate and maladaptive behavior in children, adolescents, and adults with autism spectrum disorder and intellectual disability [44]. Caregivers rate how problematic a particular behavior has been over the past week on a 4-point rating scale ranging from “not at all a problem” to “the problem is severe in degree.” The ABC-C_FXS_ specific scoring algorithm developed by Sansone and colleagues [45] assesses behavior across 6 domains including SA, Irr, and SU/L.

The CaGI-S asks caregivers to rate the overall severity of their child’s problems with social avoidance and isolation (nervousness, shyness, avoidance of other people), social interactions (communicating verbally and with body language), and irritable/disruptive behavior (temper tantrums, crying, whining) as well as overall behavior over the past week on a 4-point rating scale ranging from “no problems” to “severe problems.” The CaGI-C asks caregivers to rate the amount of change in their child’s problems compared to the beginning of the study with these behaviors on a 7-point rating scale ranging from “much better” to “much worse.”

### Safety assessments

Safety assessments included physical and neurological exams, Tanner Stage assessment, examination of skin at application sites for irritation, vital signs, 12-lead electrocardiograms, the Columbia Suicide Severity Rating Scale (children’s version), the 15-item Marijuana Withdrawal Checklist–Short Form, the Penn Physician Withdrawal Checklist, safety laboratory tests, urinalysis, seizure assessment, and adverse event (AE) monitoring.

Caregivers used a daily diary to record the presence of any skin irritation at the gel application sites, indicating whether there was redness of varying intensities. Additionally, a skin examination was conducted by the investigator at each study visit. The following Skin Irritation Check Scale was used by caregivers and investigators: 0, no erythema; 1, minimal erythema; 2, moderate erythema with sharply defined borders; 3, intense erythema with or without edema; and 4, intense erythema with edema and blistering/erosion.

### Statistical analysis

The study was designed to have 90% power to detect a − 1.3-point difference between treatments in change from baseline to week 12 ABC-C_FXS_ SA subscale score, assuming a standard deviation of 2.8 and a two-sided test at the 5% significance level. This required 102 patients per group.

The primary efficacy analysis was performed on the full analysis set (FAS), which included all patients who received ≥ 1 dose of study medication and with baseline and ≥ 1 post-baseline ABC-C_FXS_ assessments. Continuous measures including the primary endpoint were analyzed with a mixed model for repeated measures (MMRM) using an unstructured covariance matrix to estimate within-subject error, under missing at random assumption for missing data. The model included stratification variables gender and region (North America/non-North America), treatment, repeated measures for week (4, 8, 12), treatment-by-week interaction, and baseline score with baseline score-by-week interaction. Categorical response measures were analyzed with a logistic MMRM model including the same terms for stratification, treatment, week, and treatment-by-week interaction as the linear model. The hypothesis tests for the primary end point and key secondary end points were included in the strategy for strong control of the type I error probability.

Ad hoc analyses were conducted to evaluate the efficacy of ZYN002 vs placebo in patients with ≥ 90% methylation of the promoter region of the *FMR1* gene and in patients with 100% methylation of the *FMR1* gene. For analyses of outcomes in the subgroups defined by ≥ 90% methylation and 100% methylation, the MMRM statistical models described above also included the appropriate interaction terms to obtain treatment-by-subgroup interaction and inference regarding the treatment comparisons within methylation subgroups at the week 12 end point reported herein. The hypothesis tests for these treatment comparisons were exploratory in nature; therefore, nominal *P*-values are presented without adjustment for multiplicity of tests.

To aid in the interpretation of the point change on the ABC-C_FXS_ subscale scores, separate pre-planned analyses were conducted to establish meaningful change thresholds (MCTs) over 12 weeks of treatment using anchor-based methods according to the US FDA recommendation [46]. The CaGI-S and CaGI-C items served as the anchors. Change categories from baseline to week 12 were created for the CaGI-S to represent whether the severity of child’s domain-specific and overall behavior worsened or improved. Change from baseline to week 12 for each ABC-C_FXS_ subscale score was calculated for each CaGI-S change category from baseline to week 12 and for each value of the CaGI-C at week 12. Empirical cumulative distribution functions (eCDFs) of the ABC-C_FXS_ subscale scores by CaGI-S change category (Supplemental Fig. S1) and CaGI-C value (Supplemental Fig. S2) at week 12 were plotted.

MCTs were established for the ABC-C_FXS_ subscale scores by triangulation of the anchor-based analyses, eCDFs, and findings regarding meaningful change from a qualitative study in caregivers of children and adolescents with FXS [47].

Safety analyses were conducted on the safety analysis population, which included all randomized patients who received ≥ 1 dose of study medication.

All statistical analyses were performed using SAS software version 9.4 (SAS Institute).

The CONNECT-FX trial is registered on Clinicaltrials.gov (NCT03614663). This article presents analyses included in the final statistical analysis plan.

## Results

### Patients

**Fig. 2:**
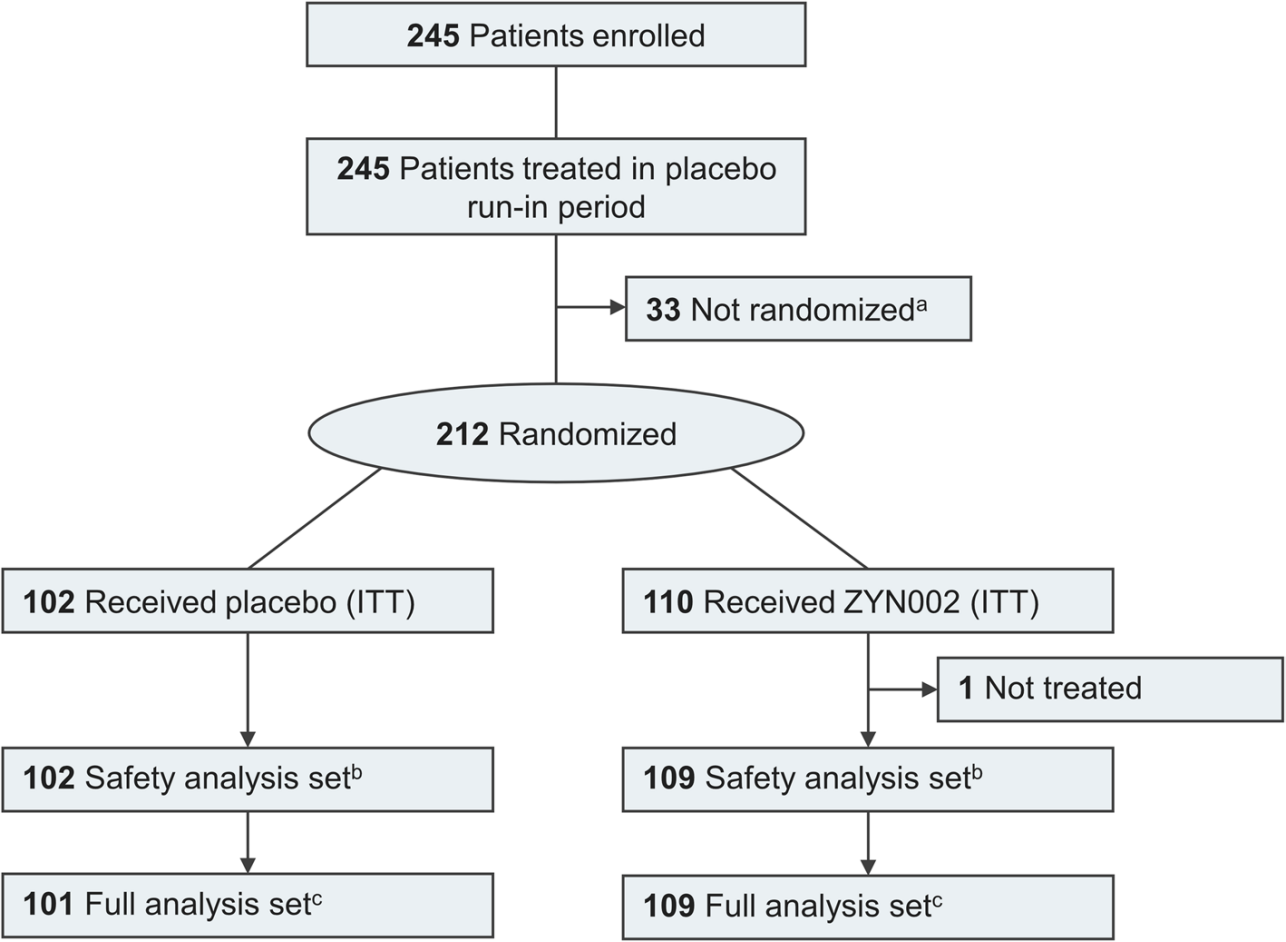
Flow diagram of participants in the CONNECT-FX trial. ^a^ indicates the following: failure to meet randomization criteria. ^b^ indicates the following: received at least one dose of double-blind treatment. ^c^ indicates the following: all patients in the safety analysis set with both a baseline and at least one post-baseline efficacy measurement

A total of 212 patients were randomized at 21 clinical sites in the USA, Australia, and New Zealand. The first patient was randomized on August 8, 2018, and the final patient visit occurred on May 15, 2020. Patient disposition is shown in Fig. 2. Of the 212 patients randomized (ZYN002, 110; placebo, 102), one did not receive study treatment. Thus, 211 patients were included in the safety analysis set. One treated patient did not have a post-baseline efficacy measure, resulting in 210 patients in the full analysis set (FAS).

### Baseline demographics and disease characteristics

The patient populations were well matched in terms of demographic and disease characteristics (Table 1). Most patients were white (78.3%) and male (75%).

**Table 1:** Baseline demographics

	**ITT analysis set (all randomized)**			** ≥ 90% methylation group**			**< 90% methylation group**		
	**Placebo** (*n* = 102)	**ZYN002** (*n* = 110)	**Total** (*N* = 212)	**Placebo** (*n* = 77)	**ZYN002** (*n* = 92)	**Total** (*n* = 169)	**Placebo** (*n* = 25)	**ZYN002** (*n* = 17)	**Total** (*n* = 42)
**Age (years)**									
Mean	9.8	9.6	9.7	9.6	9.2	9.4	10.4	12.1	11.1
Median (min, max)	10.0 (3, 17)	9.0 (3, 17)	10.0 (3, 17)	9.0 (3, 17)	9.0 (3, 17)	9.0 (3, 17)	11.0 (5, 16)	12.0 (5, 17)	11.0 (5, 17)
**Sex**—males, *n* (%)	78 (76.5)	81 (73.6)	159 (75.0)	54 (70.1)	65 (70.7)	119 (70.4)	24 (96.0)	15 (88.2)	39 (92.9)
**Weight (kg)**									
Median (min, max)	34.3 (15.6, 104.7)	36.8 (14.6, 87.0)	35.7 (14.6, 104.7)	33.9 (15.6, 104.7)	35.7 (14.6, 87.0)	35.0 (14.6, 104.7)	40.3 (21.9, 79.3)	50.0 (21.4, 66.4)	41.5 (21.4, 79.3)
**Weight category**									
> 35 kg, *n* (%)	49 (48.0)	61 (55.5)	110 (51.9)	35 (45.5)	49 (53.3)	84 (49.7)	14 (56.0)	12 (70.6)	26 (61.9)
**ADOS®-2**									
Comparison score^a^									
Median (min, max)	7.0 (1, 10)	7.0 (1, 10)	7.0 (1, 10)	8.0 (1, 10)	7.0 (1, 10)	7.0 (1, 10)	6.0 (1, 10)	7.0 (3, 10)	7.0 (1, 10)
Comparison score									
Categories,^a^ *n* (%)									
Minimal/no evidence	9 (9.1)	9 (8.4)	18 (8.7)	6 (7.9)	9 (10.0)	15 (9.0)	3 (13.0)	0	3 (7.7)
Mild	11 (11.1)	11 (10.3)	22 (10.7)	9 (11.8)	10 (11.1)	19 (11.4)	2 (8.7)	1 (6.3)	3 (7.7)
Moderate	29 (29.3)	37 (34.6)	66 (32.0)	19 (25.0)	30 (33.3)	49 (29.5)	10 (43.5)	6 (37.5)	16 (41.0)
Severe	45 (45.5)	47 (43.9)	92 (44.7)	37 (48.7)	41 (45.6)	78 (47.0)	8 (34.8)	6 (37.5)	14 (35.9)
Not applicable	5 (5.1)	3 (2.8)	8 (3.9)	5 (6.6)	0	5 (3.0)	0	3 (18.8)	3 (7.7)
Missing	3	3	6	1	2	3	2	1	3
**VABS-3**									
Overall—adaptive behavior composite scores									
*n*	100	102	202	76	84	160	24	17	41
Median (min, max)	53.5 (25, 93)	51.1 (23, 119)	52.0 (23, 119)	51.5 (24, 119)	51.5 (24, 119)	51.5 (24, 119)	60.0 (27, 84)	47.0 (23, 74)	57.0 (23, 84)

*ADOS®-2* Autism Diagnostic Observation Schedule®-2, *VABS-3* Vineland Adaptive Behavior Scales™, 3rd Edition

^a^Comparison score categories: minimal/no evidence = 1 or 2, mild = 3 or 4, moderate = 5 to 7, severe = 8 to 10

As described in the “Methods” section, ad hoc analyses were conducted to evaluate the effect of ZYN002 vs placebo in patients with ≥ 90% methylation of the promoter region of the *FMR1* gene and in patients with 100% methylation of the *FMR1* gene. The ≥ 90% methylation group represented 79.7% of the total study population. The ≥ 90% methylation patients were similar to the overall intention-to-treat (ITT) population with respect to baseline demographic and disease characteristics (Table 1). The 100% methylation group (64.8% of total study population) also had similar baseline characteristics to the ITT population and the cohort of patients with ≥ 90% methylation.

Consistent with the published literature [14, 48], patients with ≥ 90% methylation generally presented with a more severe phenotype than those who were < 90% methylated (Table 1). Baseline demographics and disease characteristics were well matched between placebo and ZYN002 in the ≥ 90% methylation group of patients (Table 1).

### Results for the primary analyses (FAS)

#### ABC-C_FXS_ subscale scores

**Fig. 3:**
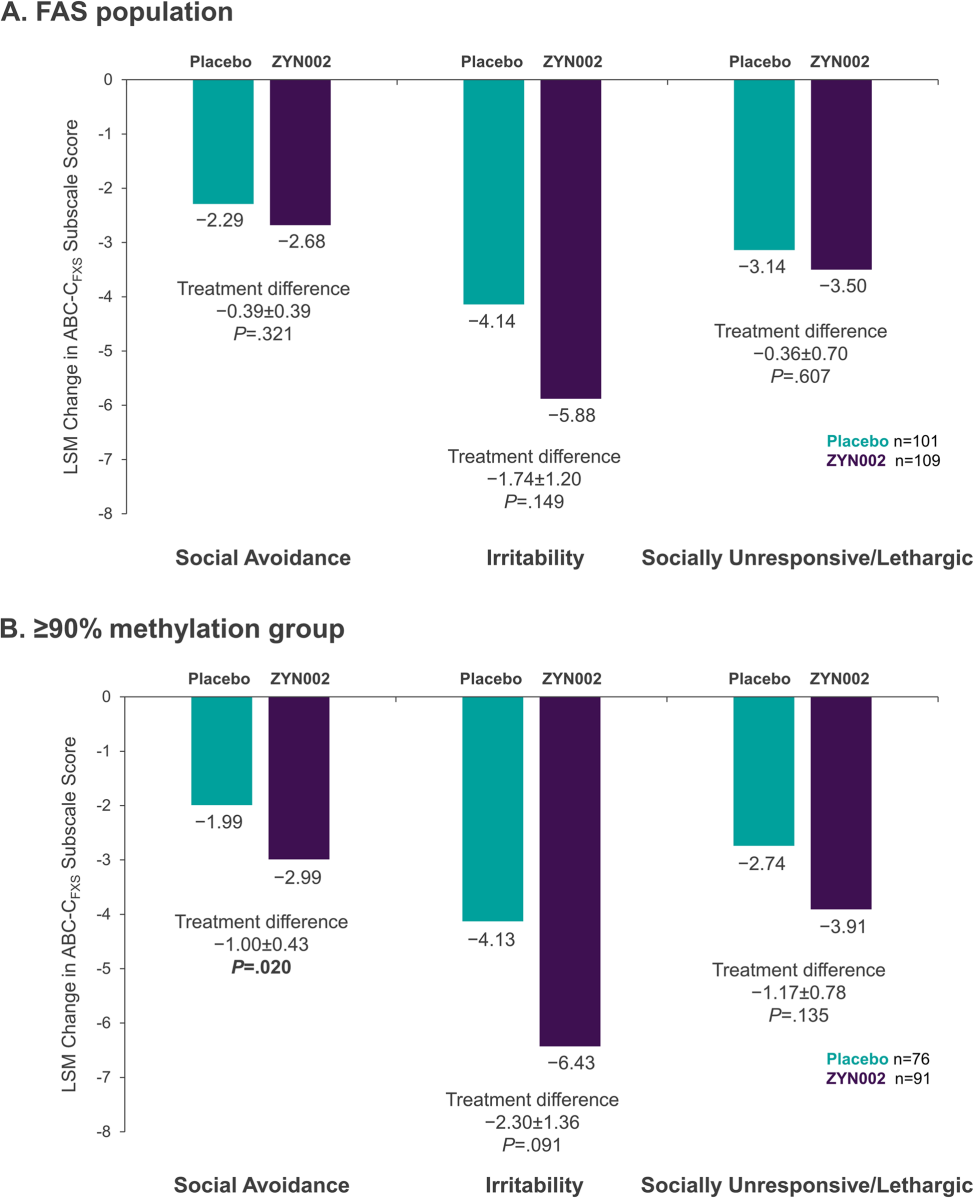
Changes from baseline in ABC-C_FXS_ subscale scores. Least square mean (LSM) changes from baseline in ABC-C_FXS_ subscale scores are shown for each treatment group along with the LSM treatment differences. **A** Results from the full analysis set. **B** Results from the patients who had ≥ 90% methylation of the promoter region of the *FMR1* gene. FAS, full analysis set

The primary efficacy end point was the change from baseline to week 12 in social avoidance (SA) as measured by the Aberrant Behavior Checklist-Community FXS specific (ABC-C_FXS_) SA subscale score [45, 49, 50]. Week 12 changes from baseline in ABC-C_FXS_ subscale scores measuring primary and secondary end points in the FAS population are shown in Fig. 3A. Although improvements in SA, irritability (Irr), and social unresponsiveness/lethargy (SU/L) (indicated by decreases in score) were greater in the ZYN002 group than in the placebo group, the differences were not statistically significant.

#### Meaningful change threshold

Mean change scores on the ABC-C_FXS_ subscales by CaGI-S baseline to week 12 change category are shown in Supplemental Table S1. For the change categories indicating improvement (i.e., 1 or 2 category change), mean change scores on the ABC-C_FXS_ SA, Irr, and SU/L subscales ranged from − 3.0 (2.96) to − 5.6 (3.06), − 8.9 (9.43) to − 13.8 (11.73), and − 3.9 (5.69) to − 7.2 (5.48) respectively for the CaGI-S domain specific and − 3.3 (4.85) to overall behavior items, respectively. A one category improvement (e.g., severe to moderate problem) on the CaGI-S were reported as a meaningful improvement in SA, Irr, and SU/L and overall behavior in the qualitative interviews [47].

Mean change scores on the ABC-C_FXS_ subscales by CaGI-C value at week 12 are presented in Supplemental Table S2. For the values indicating improvement (i.e., a little better, moderately better, and much better), mean change scores on the ABC-C_FXS_ SA, Irr, and SU/L subscales ranged from − 2.6 (3.47) to − 4.7 (3.44), − 8.6 (9.71) to − 17.4 (13.12), and − 5.1 (6.00) to − 7.8 (5.60). Any positive change on the CaGI-C was reported as meaningful or important in the caregiver cognitive interviews. As with the CaGI-S, meaningful change on the CaGI-C was considered by caregivers to imply improvement [51].

The eCDFs show clear separation between the CaGI-S change categories and the CaGI-C values representing improvement and deterioration (Supplemental Figs. 1 and 2).

Triangulating the results from the anchor-based analyses, the visual plots, and the levels of meaningful change reported by caregivers in the qualitative study, MCTs for changes from baseline to week 12 were determined to be 3 or more points on the ABC-C_FXS_ SA subscale, 9 or more points on the ABC-C_FXS_ Irr subscale, and 5 or more points on the ABC-C_FXS_ SU/L.

#### Responder analysis—ABC-C_FXS_

Responder analyses using the MCT estimates did not find a statistically significant difference between placebo and ZYN002 for SA (nominal *P* = 0.254) and SU/L (nominal *P* = 0.190) subscale scores; however, there was a trend toward statistical significance in favor of ZYN002 for Irr (nominal *P* = 0.057). The model-based estimates of percent improved at week 12 were higher for the ZYN002 group than the placebo group for all 3 subscales: 54% vs 46% for SA, 37% vs 24% for Irr, and 41% vs 32% for SU/L.

#### Clinical Global Impression, Improvement (CGI-I)

For the key secondary end point of CGI-I, the percentage of patients with improvement at week 12 was higher in the ZYN002 group compared with the placebo group, but the difference was not statistically significant. Ratings of “Very much improved,” “Much improved,” or “Minimally improved” were reported for 40.5% of the placebo group vs 46.8% of the ZYN002 group (*P* = 0.376).

#### Caregiver Global Impression-Change (CaGI-C)

The percentage of patients whose parents/caregivers indicated that their child was “a little better,” “moderately better,” or “much better” on the CaGI-C was higher for patients receiving ZYN002 compared with those receiving placebo for SA/isolation (least squares [LS] means 57.1% vs 47.6%, nominal *P* = 0.184), social interactions (61.5% vs 44.9%, nominal *P* = 0.021), irritable/disruptive behavior (48.6% vs 38.7%, nominal *P* = 0.185), and overall behavior (55.9% vs 44.9%, nominal *P* = 0.145).

### Ad hoc analyses: results in the ≥ 90% methylation group

#### ABC-C_FXS_ subscale scores

In this study, there was clear evidence of a threshold effect at 90% methylation based on a statistically significant treatment-by-subgroup interaction in change from baseline to week 12 ABC-C_FXS_ SA, (nominal *P* = 0.002); the 2 subgroups were qualitatively different, i.e., the treatment effect was reversed in the < 90% methylation group. There was no statistical evidence of a similar or greater treatment difference between subgroups below 90% methylation. The treatment effect observed in patients with ≥ 90% methylation was more than double the treatment effect observed in the overall sample.

Analysis of week 12 changes from baseline in ABC-C_FXS_ subscale scores in the ≥ 90% methylation group demonstrated statistically significant improvement in the ZYN002 patients vs placebo patients for the primary end point of SA as measured by the ABC-C_FXS_ SA subscale (treatment difference of − 1.00, nominal *P* = 0.020) (Fig. 3B). The median percent of improvement from baseline was 40.0% in the ZYN002 group vs 21.1% in the placebo group. Although statistical significance was not observed for the ABC-C_FXS_ subscales of Irr (nominal *P* = 0.091) and SU/L (nominal *P* = 0.135) in the ≥ 90% methylation group, the mean changes in score were greater for the ZYN002 group than for the placebo group for both subscales (Fig. 3B).

**Fig. 4:**
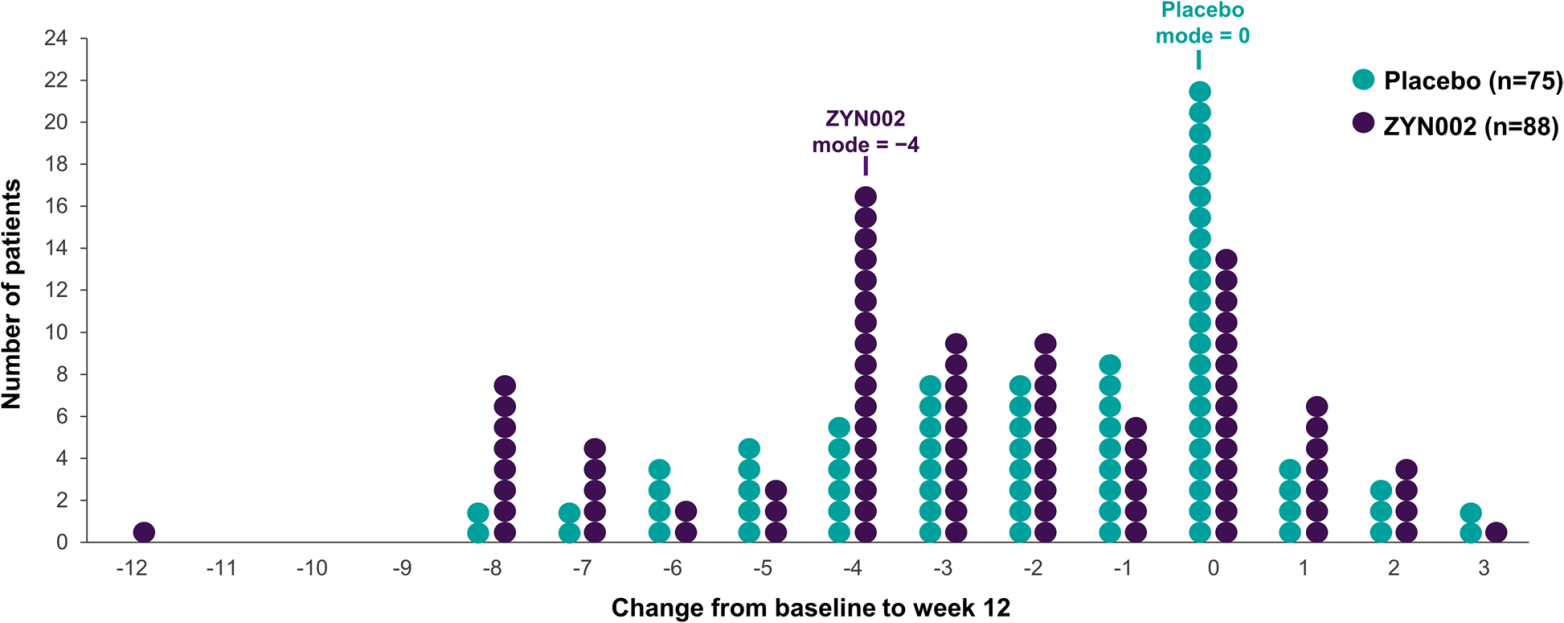
Individual patient changes from baseline to week 12 in ABC-C_FXS_ Social Avoidance subscale scores: ≥ 90% methylation group. Distribution of changes in ABC-C_FXS_ SA subscale scores at 12 weeks in the patients who had ≥ 90% methylation of the promoter region of the *FMR1* gene. Each circle represents one patient in the study. The mode for the distribution of each group is shown in the figure

The distribution of responses to ZYN002 (change from baseline in SA subscale scores) in the ≥ 90% methylation group illustrated the response to treatment, as shown in Fig. 4. In the ≥ 90% methylation group, the mode for change from baseline among patients receiving ZYN002 was − 4 vs 0 for patients receiving placebo. In the < 90% methylation group, the mode was 0 for both placebo and ZYN002; a similar number of patients in the ZYN002 treatment group improved (5/17) or worsened (6/17).

#### Responder analysis—ABC-C_FXS_

**Fig. 5:**
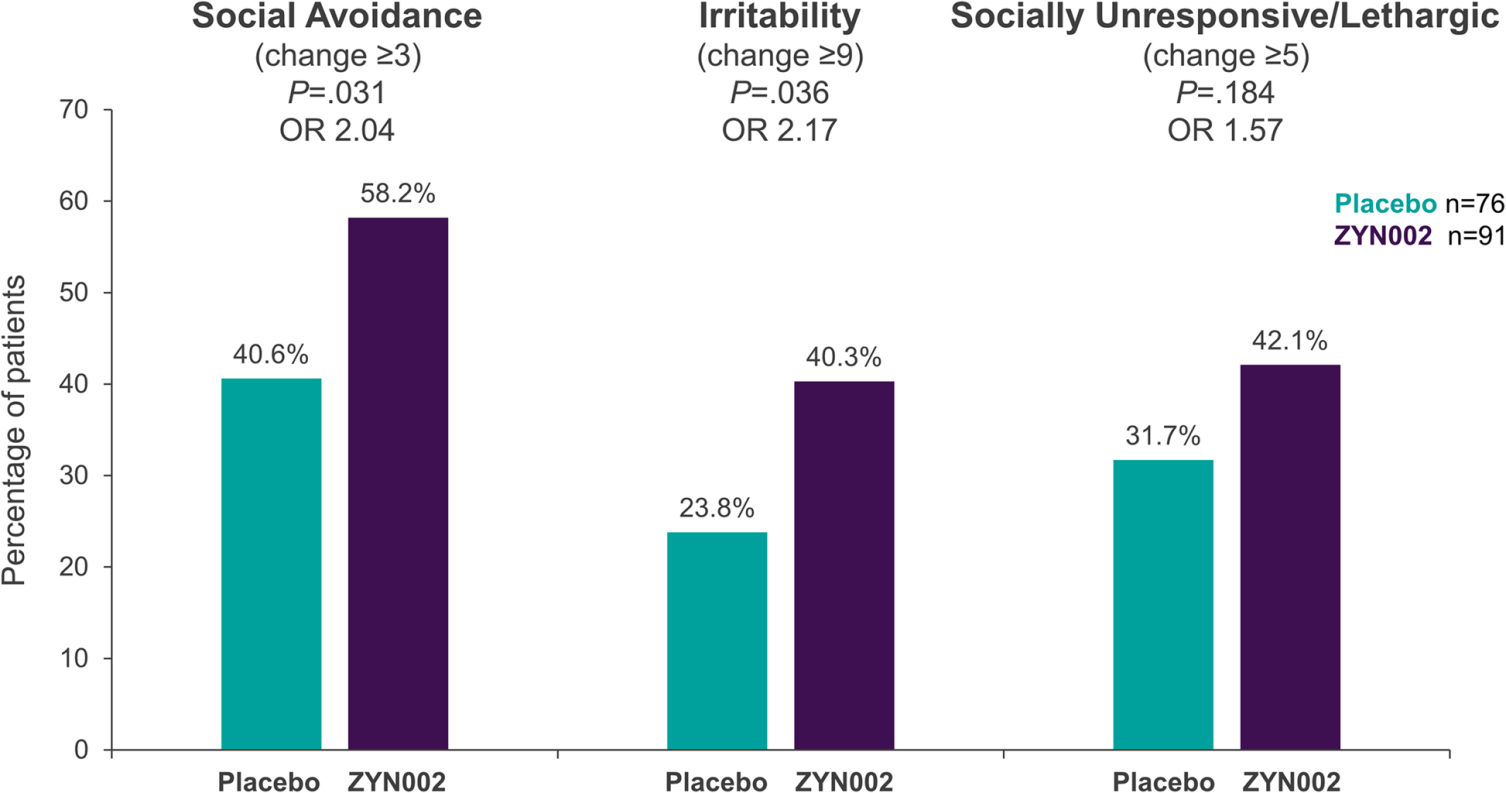
Meaningful change in ABC-C_FXS_ subscales in the ≥ 90% methylation group. Percentage of patients who experienced meaningful changes from baseline in ABC-C_FXS_ subscale scores, defined as a change of ≥ 3 for SA, ≥ 9 for Irr, or ≥ 5 for SU/L. The data represent results from the patients who had ≥ 90% methylation of the promoter region of the *FMR1* gene

In the responder analysis for clinically meaningful within-patient change for the ≥ 90% methylation group, significant differences between placebo and ZYN002 were observed for the percent of patients improved at week 12 in SA (odds ratio 2.04, nominal *P* = 0.031) and Irr (odds ratio 2.17, nominal *P* = 0.036) (Fig. 5). The model-based estimate of percent improved in the ZYN002 group was 58.2% for SA and 40.3% for Irr. The improvement at Week 12 in SA corresponded to a number needed to treat (NNT) of 5.7.

#### Clinical Global Impression, Improvement (CGI-I)

In the ≥ 90% methylation group, ratings of “Very much improved,” “Much improved,” or “Minimally improved” were reported in 37.7% of the placebo population vs 51.1% in the ZYN002 group (nominal *P* = 0.056).

#### Caregiver Global Impression-Change (CaGI-C)

**Fig. 6:**
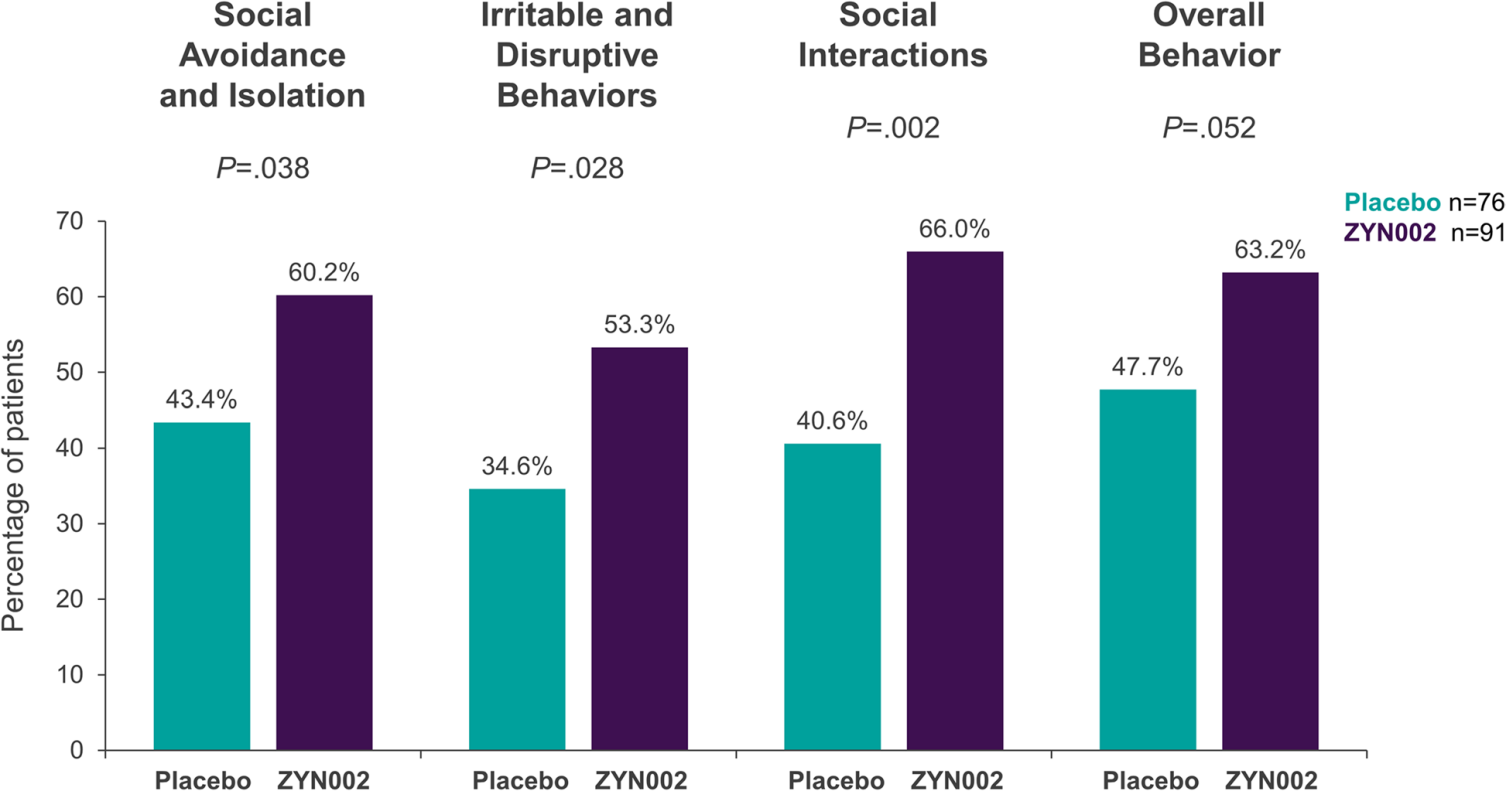
Caregiver Global Impression-Change, any improvement: ≥ 90% methylation group. Percentage of patients who recorded improvements in Caregiver Global Impression-Change. The data represent results from the patients who had ≥ 90% methylation of the promoter region of the *FMR1* gene

In the ≥ 90% methylation group, the percentage of patients whose parents/caregivers indicated that their child was “a little better,” “moderately better,” or “much better” was statistically significantly higher for patients receiving ZYN002 compared with those receiving placebo for all 3 items in CaGI-C, SA/isolation, social interactions, and irritable/disruptive behavior (all nominal *P* < 0.05) and neared significance for overall behavior (nominal *P* = 0.052) (Fig. 6).

### Ad hoc analysis: results in the 100% methylation group

An ad hoc analysis of patients with 100% methylation of the promoter region of the *FMR1* gene was conducted to further explore the impact of complete methylation on response to ZYN002. This analysis revealed a further increase in treatment effect. The effect sizes were − 0.14 for the primary analysis, − 0.36 for the ≥ 90% methylation group, and − 0.39 for 100% methylation group. In patients with 100% methylation, ZYN002 was associated with 40% median improvement in the ABC-C_FXS_ SA (treatment difference of − 1.08, nominal *P* = 0.027). Statistically significant effects were also observed for responder analyses for clinically meaningful change in ABC-C_FXS_ SA (≥ 3 points; 56% for ZYN002 vs 37% for placebo [nominal *P* = 0.030]) and in the CaGI-C for any improvement in social interaction (63% for ZYN002 vs 37% for placebo [nominal *P* = 0.005]) and irritable/disruptive behaviors (54% for ZYN002 vs 33% for placebo [nominal *P* = 0.027]).

### Safety

Approximately half (54.0%) of the 211 patients included in the safety analysis population experienced at least one treatment-emergent adverse event (TEAE). The frequency of TEAEs was similar for the placebo and ZYN002 treatment groups (50.0% and 57.8%, respectively) (Table 2). In the ≥ 90% methylation group, 55.4% of patients in the safety analysis population (*n* = 168) experienced at least one TEAE (placebo: 51.9%; ZYN002: 58.2%). In the < 90% methylation group, 47.6% experienced at least one TEAE (placebo: 44.0%; ZYN002: 52.9%). All TEAEs were mild or moderate in severity.

**Table 2:** Treatment-emergent adverse events occurring in > 1% of patients (safety analysis set)

Adverse event, *n* (%)	Placebo (*n* = 102)	ZYN002 (*n* = 109)
Patients with at least 1 TEAE	51 (50)	63 (57.8)
Upper respiratory tract infection	7 (6.9)	15 (13.8)
Nasopharyngitis	9 (8.8)	10 (9.2)
Vomiting	6 (5.9)	8 (7.3)
Pyrexia	7 (6.9)	5 (4.6)
Application site pain	1 (1.0)	7 (6.4)
Diarrhea	0 (0)	5 (4.6)
Gastroenteritis	1 (1.0)	7 (6.4)
Pharyngitis streptococcal	2 (2.0)	3 (2.8)
Rhinorrhea	2 (2.0)	2 (1.8)
Cough	0 (0)	3 (2.8)
Rash	1 (1.0)	2 (1.8)
Skin abrasion	1 (1.0)	2 (1.8)
Viral upper respiratory tract infection	2 (2.0)	1 (0.9)

There were no serious adverse events (SAEs) or severe TEAEs reported during the study. One patient in the placebo group discontinued study treatment due to a TEAE (stereotypy). The most frequently reported TEAEs in either treatment group were upper respiratory tract infections. Psychiatric disorder TEAEs, primarily symptoms of FXS, were reported for 5 (4.9%) patients in the placebo group (anxiety, impulsive behavior, irritability, staring, stereotypy: 1 patient each) and 2 (1.8%) patients in the ZYN002 group (aggression, bruxism: 1 patient each).

Treatment-related TEAEs were reported for 4 (3.9%) patients in the placebo group (total of 6 events) and 11 (10.1%) patients in the ZYN002 group (total of 14 events). In the ≥ 90% methylation group, 8.3% had at least one treatment-related TEAE (placebo: 5.2%; ZYN002: 11.0%). In the < 90% methylation group, 2.4% experienced at least one treatment-related TEAE. The most common treatment-related TEAE was application site pain, reported in 1 (1.0%) placebo-treated patient and 7 (6.4%) ZYN002-treated patients. Application site pain was mild except for 2 events of moderate severity in the ZYN002 group. All other application site TEAEs reported in the ZYN002 group (application site dryness and application site pruritus in 1 patient, application site rash in 1 patient, and application site reaction in 1 patient) were of mild severity. Application site urticaria of moderate severity was reported for 1 patient in the placebo group.

Regarding skin assessments, 97% and 89% of individual daily diary skin irritation scores assessed by caregivers were “0,” no erythema, for placebo- and ZYN002-treated patients, respectively. The percentage of patients with a score of “2,” moderate erythema with sharply defined borders, recorded by caregivers during months 1, 2, and 3 of treatment, respectively, were 2.9%, 2.0%, and 2.0% for the placebo group and 17%, 5.6%, and 1.9% for the ZYN002 group. The only scores of “3,” intense erythema with or without edema, were recorded during month 1 of randomized treatment for 1 patient (1%) in the placebo group and 3 patients (2.8%) in the ZYN002 group. There were no scores of “4,” intense erythema with edema and blistering/erosion, reported by caregivers for any patients.

The highest skin irritation score assigned by investigators was a score of “2” recorded for 2 of 104 (1.9%) patients in the ZYN002 group at week 4, 1 of 98 (1.0%) at week 8, and for 1 of 98 (1.0%) patients in the placebo group at weeks 8 and 12.

Changes from baseline in laboratory values for chemistry and hematology were comparable between the placebo and ZYN002 treatment groups, and there were no clinically relevant abnormalities in either group. There were no clinically significant changes in liver function tests reported in any patient. In both treatment groups, overall changes from baseline in vital signs and ECG parameters were minimal and not clinically significant.

## Discussion

### Overview

CONNECT-FX was the single largest double-blind, randomized, placebo-controlled trial performed in FXS. The number of children and adolescents included in the ≥ 90% methylation group (169 patients), alone, was as large as study populations in most other clinical trials of FXS.

In the ≥ 90% methylation group, ZYN002 was superior to placebo in multiple analyses. In addition, an analysis of responder thresholds for meaningful within-patient behavioral change on the ABC-C_FXS_ revealed specific thresholds for the ABC-C_FXS_ subscales, indicating that the ABC-C_FXS_ is well suited for assessing meaningful changes in these behaviors [47]. In the ZYN002 group, the LS mean change from baseline at week 12 in the ABC-C_FXS_ SA subscale (− 2.99) met the threshold for meaningful within-patient change [47]. The proportions of patients attaining a threshold of meaningful within patient change in SA and Irr were significantly greater with ZYN002 vs placebo. There was a statistically significantly higher percentage of patients reported as improved based on caregiver reported global impression of change for SA, social interaction, and irritable behaviors with ZYN002 vs placebo. Parents/caregivers consider any improvement in this measure to be important, highlighting the potential benefit of ZYN002 in this patient population. These results demonstrate the consistency of the effect of ZYN002 in the treatment of behavioral symptoms associated with FXS in patients with ≥ 90% methylation of the *FMR1* gene. A confirmatory phase 3, randomized, controlled trial (ZYN2-CL-033, RECONNECT; NCT04977986) is being conducted in children and adolescent patients with FXS.

ZYN002 was well tolerated. There were no SAEs reported during the study. All TEAEs (any event, whether unrelated or related to study drug) were mild or moderate. There were fewer psychiatric disorder TEAEs reported in the ZYN002 patients compared with the placebo patients, suggesting that ZYN002 treatment may have reduced the periodic exacerbations of symptoms that are typically associated with FXS (i.e., anxiety, impulsive behavior, irritability, staring, and stereotypy). There were no apparent differences in safety or tolerability based on the methylation status of patients. No clinically significant changes in liver function tests were reported in this trial. Liver enzyme elevations have been documented in previous clinical trials with oral formulations of cannabidiol [52, 53]. The lack of liver function elevations in the CONNECT-FX trial may be due to the transdermal route of administration of cannabidiol in ZYN002.

### Biologically identifiable population

As described above, improvements in ABC-C_FXS_ SA subscale scores were greater in the ZYN002 group than in the placebo group. Although the differences were not statistically significant in the FAS, the differences were statistically significant in the patients with ≥ 90% methylation of the promoter region of the *FMR1* gene, with the greatest treatment effect seen in those patients with 100% methylation of the *FMR1* gene promoter region. Thus, CONNECT-FX appears to provide evidence that identifies a biologically identifiable and clinically responsive population of patients affected by FXS who are defined by both full mutation and ≥ 90% methylation of the *FMR1* gene. CONNECT-FX may also support the hypothesis that targeted intervention with cannabidiol, intended to modulate EC system dysfunction in FXS, can produce clinically relevant improvement in behavioral symptoms of FXS.

Patients with ≥ 90% methylation of the promoter region of the *FMR1* gene represented 80% of the patients in the CONNECT-FX trial and are estimated to represent approximately 70% of patients with FXS. Previous studies demonstrated that persons with FXS and a FM may not have complete silencing of the *FMR1* gene and as such may still be producing FMRP [9, 54]. The patients in CONNECT-FX who had FXS with an FM with ≥ 90% methylation may, therefore, represent a population with almost complete or complete silencing of the *FMR1* gene with little to no FMRP production and, further, may represent a population that is most responsive to ZYN002. This may help explain why those with < 90% methylation did not respond as well as those with ≥ 90% methylation. We could hypothesize that those who are mosaic with unmethylated alleles, whether in the premutation or in the full mutation range, produce mRNA and FMRP that could potentially interfere with the putative positive effects of ZYN002.

While girls with ≥ 90% methylation likely produce more FMRP than boys due to the presence of a normal allele, girls with greater methylation of the affected allele and/or a low activation ratio are more phenotypically similar to boys; for example, like boys, significant cognitive impairment occurs in girls with significant methylation [55]. Withdrawn and anxious behaviors have also been reported to be greater in girls with FXS [56] and in girls in the general population [57]. The underlying physiology of the EC system in girls may also lead to increased responsiveness in girls [58]. Taken together, these findings may explain why the girls with ≥ 90 methylation responded to ZYN002 despite the likely presence of more FMRP than boys.

### Study limitations

Because the study was limited to children and adolescents with FXS, the study results may not necessarily be generalizable to adult patients with FXS. Moreover, the effects of ZYN002 on the outcomes measures was seen predominantly in the patients with ≥ 90% methylation in *FMR1* with less pronounced effects in patients with < 90% methylation in *FMR1*. While the results of this trial suggest a lower response for patients with < 90% methylation in *FMR1*, the sample size of that population was small and as such a definitive conclusion cannot be drawn in regard to the effect of ZYN002 in that population. As such, the study results may therefore not be generalizable to patients with < 90% methylation in *FMR1*. The primary endpoint, the SA subscale of the ABC-C_FXS_, includes only 4 items and may therefore not measure all aspects of anxiety and social anxiety in patients with FXS that may be affected by ZYN002. A small number of patients (*n* = 13) were enrolled who had ABC-C_FXS_ SA subscale scores of 2 to 3 at baseline (plus Irr score ≥ 18; as described in the “Methods” section), and it is possible the inclusion of these patients may have limited the ability to detect improvement in SA in those patients. In addition, the use of fixed, weight-based dosing for ZYN002 led to various drug doses on a mg/kg basis, especially in higher weight individuals. A broader dose range, up to 750 mg/day in patients weighing more than 50 kg, has been incorporated in a follow-up study to reconfirm the results seen in CONNECT-FX.

## Conclusions

In this trial, ZYN002 was well tolerated in patients with FXS and demonstrated evidence of efficacy with a favorable benefit risk relationship in patients with ≥ 90% methylation of the promoter region of the *FMR1* gene, in whom gene silencing is most likely, with little or no FMRP production, and the impact of FXS is typically most severe.

## Supplementary Information

**Additional file 1: Supplemental Table S1.** Change in ABC-C_FXS_ Social Avoidance, Irritability, and Socially Unresponsive/Lethargic Subscale Scores by CaGI-S Change Categories. **Supplemental Table S2.** Change in ABC-C_FXS_ Social Avoidance, Irritability, and Socially Unresponsive/Lethargic Subscale Scores by CaGI-C Week 12 Values. **Supplemental Figure S1.** Empirical cumulative distribution function curves of change in the ABC-C_FXS_ SA, Irritability, and SU/L subscale scores by change in the CaGI-S domain-specific and overall behavior scores. **Supplemental Figure S2.** Empirical cumulative distribution function curves of change in the ABC-C_FXS_ SA, Irritability, and SU/L subscale scores by change in the CaGI-C domain-specific and overall behavior scores.

## Data Availability

All data generated or analyzed during this study are included in this published article.

## Abbreviations

2-AG: 2-Arachidonoylglycerol
ABC-C_FXS_: Aberrant Behavior Checklist–Community Edition FXS
ADOS®-2: Autism Diagnostic Observation Schedule®-2
AE: Adverse event
AEA: Anandamide
ASD: Autism spectrum disorder
CaGI-C: Caregiver Global Impression-Change
CGG: Cytosine, guanine, and guanine
CGI-I: Clinical Global Impression, Improvement
CGI-S: Clinical Global Impression-Severity
CNS: Central nervous system
DAGL: Diacylglycerol lipase
EC: Endocannabinoid
FAS: Full analysis set
FM: Full mutation
FXS: Fragile X syndrome
Irr: Irritability
IRT: Interactive response system
ITT: Intention-to-treat
LS: Least squares
MMRM: Mixed model for repeated measures
NNT: Number needed to treat
SA: Social avoidance
SAEs: Serious adverse events
SU/L: Social Unresponsiveness/Lethargy
TEAE: Treatment-emergent adverse event
THC: Tetrahydrocannabinol
ULN: Upper limit of normal
VABS-3: Vineland Adaptive Behavior Scales™, 3rd Edition
