# Supplementary material for: A randomized, controlled trial of ZYN002 cannabidiol transdermal gel in children and adolescents with fragile X syndrome (CONNECT-FX)
Source: J Neurodev Disord. 2022 Nov 25;14:56. doi: 10.1186/s11689-022-09466-6 (PMC9700889; doi:10.1186/s11689-022-09466-6)

**CONNECT-FX Supplemental Tables and Figures**

**Berry-Kravis et al. 2022**

**Supplemental Table S1.** Change in ABC-C_FXS_ Social Avoidance, Irritability, and Socially Unresponsive/Lethargic Subscale Scores by CaGI-S Change Categories

| **ABC-C_FXS_** | **CaGI-S Change Category^a^** | | | | |
| --- | --- | --- | --- | --- | --- |
|  | **−2**  **Mean (SD) [n]** | **−1**  **Mean (SD) [n]** | **0**  **Mean (SD) [n]** | **1**  **Mean (SD) [n]** | **2**  **Mean (SD) [n]** |
| *Social Avoidance* | | | | | |
| CaGI-S DS | −5.6 (3.06) [17] | −3.0 (3.0) [60] | −1.5 (2.44) [96] | −0.2 (1.91) [2] | N/A [0] |
| CaGI-S OB | −3.3 (4.85) [9] | −3.6 (2.64) [47] | −1.8 (2.85) [110] | −0.9 (2.22) [27] | N/A [0] |
| *Irritability* | | | | | |
| CaGI DS | −13.8 (11.73) [15] | −9.8 (9.7) [55] | −2.5 (6.34) [90] | 1.2 (6.85) [29] | −3.8 (6.19) [4] |
| CaGI OB | −10.1 (17.06) [9] | −8.9 (9.43) [47] | −3.9 (8.01) [110] | −0.4 (4.14) [27] | N/A [0] |
| *Socially Unresponsive/Lethargic* | | | | | |
| CaGI DS | −7.2 (5.48) [13] | −5.4 (6.46) [55] | −2.3 (5.01) [98] | −1.7 (4.14) [23] | −5.5 (2.08) [4] |
| CaGI OB | −3.9 (5.69) [9] | −6.8 (6.92) [47] | −2.7 (4.76) [110] | −0.9 (3.55) [27] | N/A [0] |

Abbreviations: ABC-C_FXS_, Aberrant Behavior Checklist in Fragile X Syndrome; CaGI-S, Caregiver Global Impression of Severity; DS, domain specific; N/A, not applicable; OB, overall behavior.

^a^Difference between CaGI-S baseline value and CaGI-S week 12 value. Higher change categories are associated with worening behavior.

**Supplemental Table S2.** Change in ABC-C_FXS_ Social Avoidance, Irritability, and Socially Unresponsive/Lethargic Subscale Scores by CaGI-C Week 12 Values

|  | **CaGI-C Week 12 Value** | | | | | | | |
| --- | --- | --- | --- | --- | --- | --- | --- | --- |
| **ABC-C_FXS_** | **3**  **Mean (SD) [n]** | **2**  **Mean (SD) [n]** | **1**  **Mean (SD) [n]** | **0**  **Mean (SD) [n]** | **−1**  **Mean (SD) [n]** | **−2**  **Mean (SD) [n]** | **−3**  **Mean (SD) [n]** |  |
| *Social Avoidance* | | | | | | | | |
| CaGI-C DS | 4.2 (2.77) [9] | −4.4 (2.90) [32] | −2.6 (3.47) [55] | −0.8 (2.23) [95] | 1. (1.23) [5] | −2.5 (3.11) [4] | N/A [0] |  |
| CaGI-C OB | −4.7 (3.44) [13] | −4.0 (3.25) [24] | −2.9 (2.95) [64] | −0.7 (2.39) [83] | 0.6 (1.37) [11] | −3.2 (1.92) [5] | N/A [0] |  |
| *Irritability* |  |  |  |  |  |  |  |  |
| CaGI-C DS | −17.4 (13.13) [12] | −12.4 (8.74) [18] | −9.0 (9.62) [41] | −3.5 (7.71) [105] | −2.7 (6.00) [16] | 1.0 (4.62) [7] | 1.0 [1] |  |
| CaGI-C OB | −14.1 (11.79) [13] | −10.3 (9.99) [24] | −8.6 (9.71) [64] | −2.6 (7.15) [83] | −0.4 (3.67) [11] | −0.4 (7.47) [5] | N/A [0] |  |
| *Socially Unresponsive/Lethargic* | | | | | | | | |
| CaGI-C DS | −7.8 (5.60) [16] | −7.3 (6.26) [24] | −5.1 (6.00) [57] | −1.7 (4.27) [96] | −3.8 (6.46) [6] | 0.0 [1] | N/A [0] |  |
| CaGI-C OB | −6.8 (5.95) [13] | −7.1 (6.78) [24] | −5.4 (5.65) [64] | −1.6 (4.51) [83] | −2.1 (2.51) [11] | −3.2 (6.72) [5] | N/A [0] |  |

Abbreviations: ABC-C_FXS_, Aberrant Behavior Checklist in Fragile X Syndrome; CaGI-C, Caregiver Global Impression of Change; DS, domain specific; N/A, not applicable; OB, overall behavior.

**Supplemental Figure S1.** Empirical cumulative distribution function curves of change in the ABC-C_FXS_ SA, Irritability, and SUL subscale scores by change in the CaGI-S domain-specific and overall behavior scores


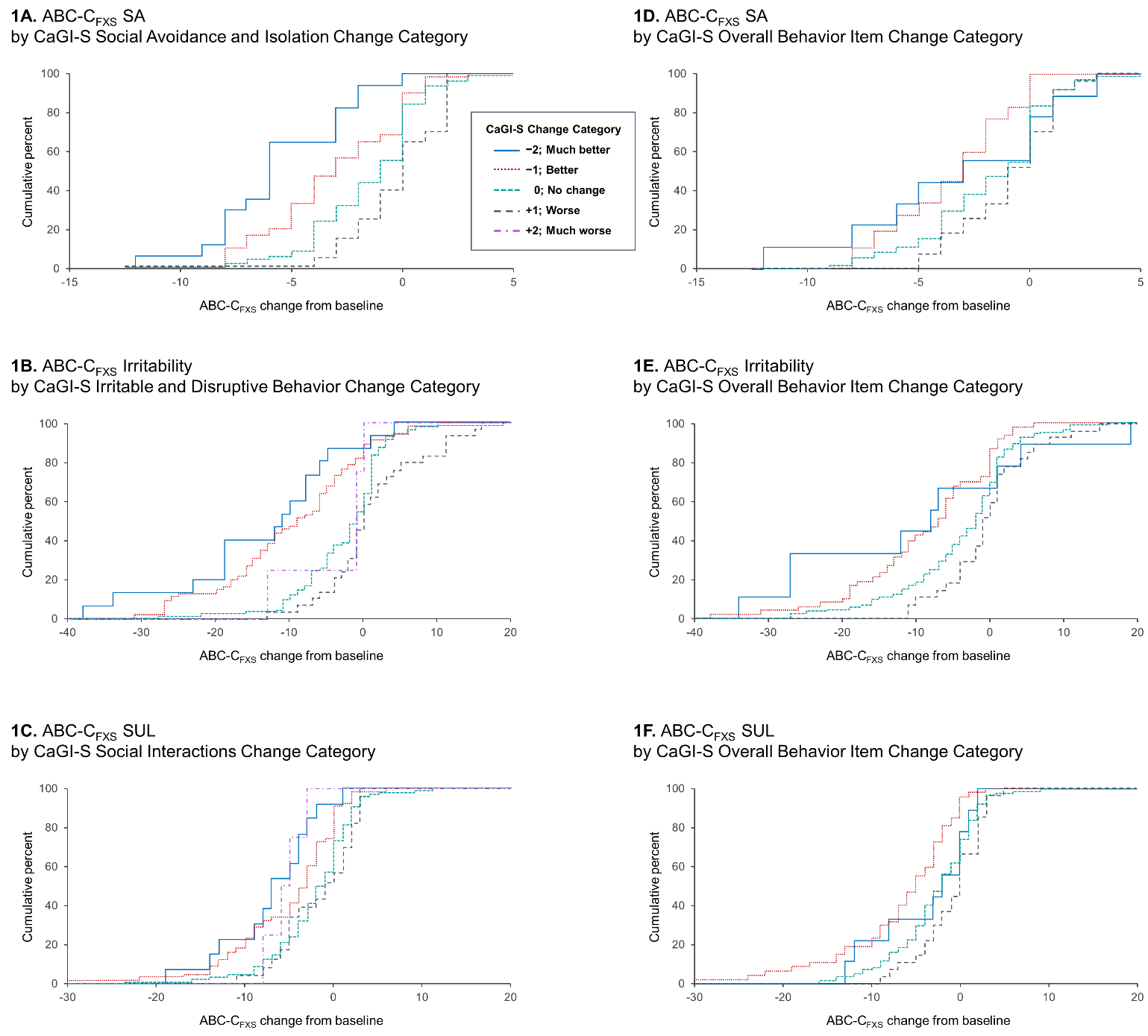


**Supplemental Figure S2.** Empirical cumulative distribution function curves of change in the ABC-C_FXS_ SA, Irritability, and SUL subscale scores by change in the CaGI-C domain-specific and overall behavior scores


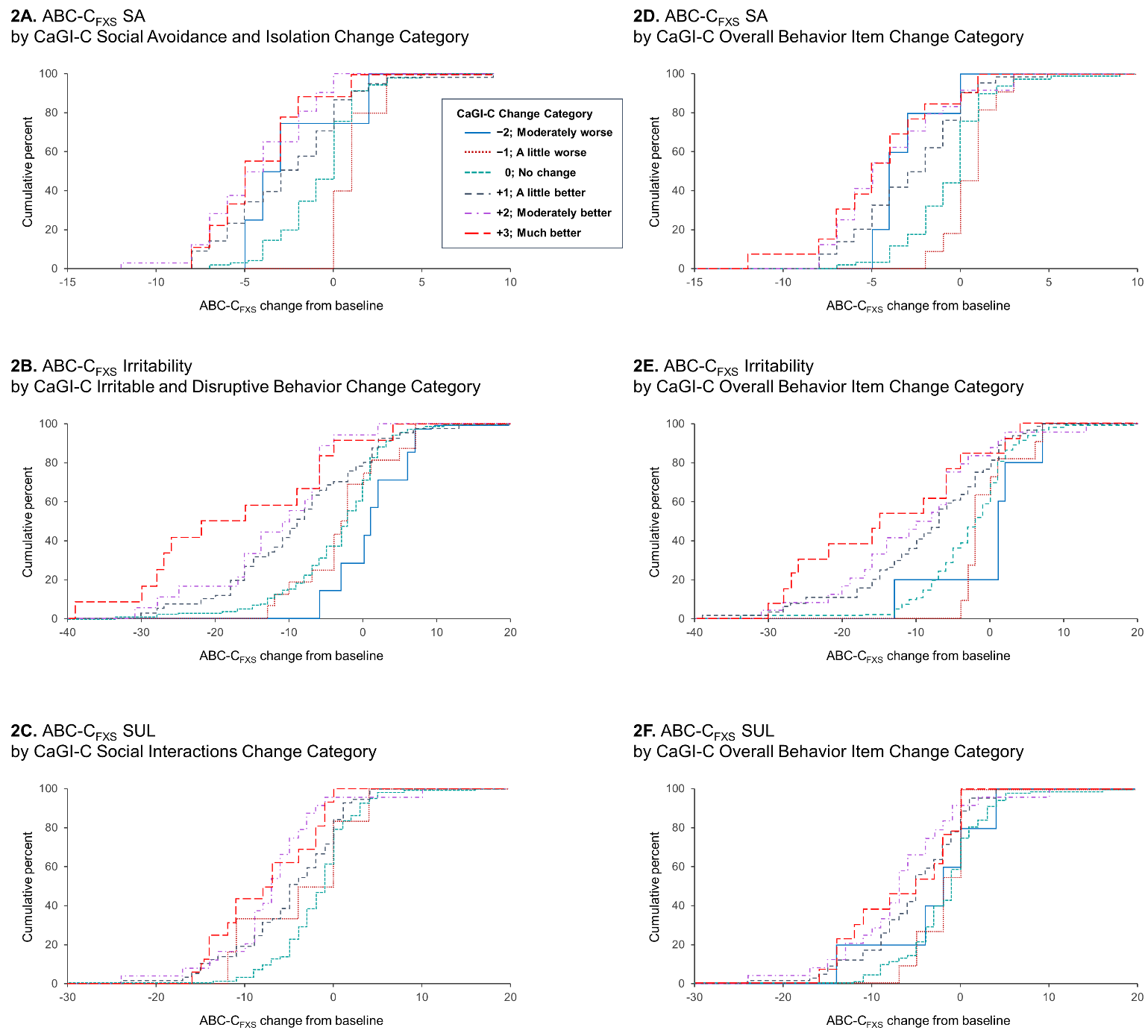

Supplement: Supplementary file 1 — Additional file 1: Supplemental Table S1. Change in ABC-CFXS Social Avoidance, Irritability, and Socially Unresponsive/Lethargic Subscale Scores by CaGI-S Change Categories. Supplemental Table S2. Change in ABC-CFXS Social Avoidance, Irritability, and Socially Unresponsive/Lethargic Subscale Scores by CaGI-C Week 12 Values. Supplemental Figure S1. Empirical cumulative distribution function curves of change in the ABC-CFXS SA, Irritability, and SU/L subscale scores by change in the CaGI-S domain-specific and overall behavior scores. Supplemental Figure S2. Empirical cumulative distribution function curves of change in the ABC-CFXS SA, Irritability, and SU/L subscale scores by change in the CaGI-C domain-specific and overall behavior scores. [file 11689_2022_9466_MOESM1_ESM.docx]
